# Impact of retrograde flexible ureteroscopy and intracorporeal lithotripsy on kidney functional outcomes

**DOI:** 10.1590/S1677-5538.IBJU.2014.0402

**Published:** 2015

**Authors:** Nicolas Hoarau, Francois Martin, Souhil Lebdai, Denis Chautard, Thibaut Culty, Abdel Rahmene Azzouzi, Pierre Bigot

**Affiliations:** 1 Département d'urologie, Hôpital universitaire d'Angers, Angers, France

**Keywords:** kidney stones, holmium laser lithotripsy, renal function, retrograde intrarenal surgery, urolithiasis

## Abstract

**Objective:**

The aim of the study was to evaluate renal function and to identify factors associated with renal function deterioration after retrograde intrarenal surgery (RIRS) for kidney stones.

**Materials and Methods:**

We retrospectively analyzed patients with renal stones treated by RIRS between January 2010 and June 2013 at a single institute. We used the National Kidney Foundation classification of chronic kidney disease (CKD) to classify Glomerular Filtration Rate (GFR) in 5 groups. The baseline creatinine level was systematically pre-operatively and post-operatively evaluated. All patients had a creatinine blood measurement in June 2013. A change toward a less or a more favorable GFR group following RIRS was considered significant.

**Results:**

We included 163 patients. There were 86 males (52.8%) and 77 females (47.3%) with a mean age of 52.8±17 years. After a mean follow-up of 15.5±11.5 months, median GFR was not significantly changed from 84.3±26.2 to 84.9±24.5 mL/min (p=0.675). Significant renal function deterioration occurred in 8 cases (4.9%) and significant renal function amelioration occurred in 23 cases (14.1%). In univariate analysis, multiple procedures (p=0.023; HR: 5.4) and preoperative CKD (p=0.011; HR: 6.8) were associated with decreased renal function. In multivariate analysis these factors did not remain as predictive factors.

**Conclusion:**

Stone management with RIRS seems to have favorable outcomes on kidney function; however, special attention should be given to patients with multiple procedures and preoperative chronic kidney disease.

## INTRODUCTION

Retrograde intrarenal surgery (RIRS), combined with Holmium laser lithotripsy is widely applied for the management of intra-renal stones. For large or multiple stones, RIRS may represent an alternative therapy to percutaneous nephrolithotomy (PNL) even if multiple procedures are often required (1, 2). It is accepted that stone removal can improve renal function; however, a stone-removing procedure may negatively impact the kidney parenchyma (3). Recently, El-Tabey et al. reported that Percutaneous Nephrolithotomy (PNL) for calculi in solitary kidneys provided significant improvement in renal function at long-term follow-up (4).

To date, no study has evaluated the impact of RIRS on renal function. Nevertheless, in case of RIRS, the flexible ureteroscope is introduced into the upper urinary tract collecting system under water pressure. Irrigation during endoscopy cool the tip of energy-delivering devices and helps to maintain a clear visual field by displacing blood, stone fragments, and cellular debris. However, this leads to prolong renal calices distension. Moreover, delivering laser energy next to or directly onto renal tissue may cause damage to the renal papillae. Thus, one could advocate neither prolonging nor multiplying the surgical procedure to avoid renal function deterioration and to decrease post-operative complications. The type and the time of post-operative ureteral catheterization could also protect from renal dysfunction.

The aim of this study was to evaluate renal function and identify factors associated with renal function deterioration or amelioration after RIRS.

## MATERIALS AND METHODS

### 

#### Patients

After approval from an institutional review board, we retrospectively analyzed 163 patients undergoing 205 RIRS for intra-renal stones between January 2010 and June 2013 in an academic department of Urology. All patients with a unique radio-opaque stone were previously treated by at least one procedure of Shock Wave Lithotripsy (SWL) without result. Patients were pre and post-operatively evaluated by computed tomography. All procedures were conducted under sterile urine and verified by a urine culture seven days before the RIRS. Confirmed urinary infection or bacteriurias were systematically treated during at least five days with adapted antibiotherapy. We recorded patient age, gender, body mass index, number of stones, diameter of the largest stone, cumulative stone diameter, and stone composition. The perioperative parameters analyzed were the use of pre-operative stenting, the mean operative time, the use of a UAS (Ureteral Access Sheath), the presence of a post-operative ureteral stent or a JJ-stent, the number of procedures and the mean follow-up. The stone free (SF) status was defined when no residual fragments (<2mm) were seen on a non-contrast enhanced tomography performed 1 to 2 months after the last retrograde flexible ureteroscopy session. We performed another RIRS in case of significant persistent stone (>4mm) on the post-operative tomography. RIRS were performed between 2 and 3 months after the previous procedure.

#### Surgical procedure

The procedures were performed by 10 different surgeons from the same institution. All procedures were done under general anesthesia in a standard flexible ureteroscopy installation. A 0.035 inch polytetrafluoroethylene coated wire was placed in the upper urinary tract under visual and fluoroscopic control through a rigid cystoscope. A safety wire was routinely used. The use of a UAS (Ureteral Access Sheath with AQ, Cook Medical, Spencer, USA) was done under the assessment of the surgeon, as well as its insertion method. When no UAS was used, the flexible ureteroscope (Flex-X2 TM, Karl Storz endocopy, Tuttlingen, Germany) was inserted in a monorail way over the second wire. Normal saline irrigation was performed at a pressure of 60 cm H_2_O through the same channel used for working instruments. If necessary a transient water pressure was carried out using a hand pump. A holmium–YAG laser was used at an energy varying between 0.6 to 0.8 Joules, and at a frequency of 8 to 10 Hertz. A 270μm or 400μm laser fibre was used for delivering laser energy. At the end of the procedure a JJ stent or a ureteral stent was inserted under the assessment of the surgeon.

#### Renal function evaluation

The evaluation of the Glomerular Filtration Rate (GFR) was derived from the Modification of Diet in Renal Disease (MDRD) study group equation (5). The baseline creatinine level was systematically pre-operatively and post-operatively (day one) evaluated. All patients had a creatinine blood measurement in June 2013.

We used the National Kidney Foundation classification of chronic kidney disease (CKD) which classifies estimated GFR in the following ranges: at least 60, 45 to 59 (stage 3a), 30 to 44 (stage 3b), 15 to 29 (stage 4), and less than 15 mL per minute per 1.73m^2^ (stage 5) (6). A change toward a less or a more favorable GFR group following surgery was considered significant.

### Statistical analysis

Pair t-test was used for comparisons of GFR before and after RIRS. Independent-sample t-test and chi-square tests were used for comparisons of means and proportions, respectively. Univariate and multivariate regression models were used to assess the influence of different variables on renal function outcomes. Only factors that were significant in univariate analysis were considered for multivariate analysis. All tests were done using SPSS^®^ version 10.

## RESULTS

### 

#### Patient and stone characteristics

We included 163 patients. There were 86 males (52.8%) and 77 females (47.3%) with a mean age of 52.8±17 years. The mean BMI was 26.2±5.9 cm/kg. Patients presented a GFR greater than 60 mL/min/1.73m^2^ in 128 (78.5%) cases. Patients presented 3a, 3b and 4 preoperative CKD stage in 27 (16.6%), 7 (4.3%) and 1 (0.6%) cases, respectively. The mean GFR was 84.30±26.2 mL/min/1.73m^2^.

Multiple stones were present in 73 patients (44.7%). The mean diameter of the largest stone was 12.9±5.7mm, and the cumulative stone diameter was 15±8.6mm. Stone composition was calcium oxalate monohydrate, calcium oxalate dehydrate, uric acid, carbapatite, and unknown in 56 (34%), 8 (5%), 13 (8%), 27 (16.6%) and 86 (53%) cases, respectively. A pre-operative stent was inserted in 46 (63.8%) cases. The mean operative time was 96.4±40.78 minutes. A ureteral access sheath was used in 144 (88.3%) procedures and a postoperative ureteral JJ stent was left in 115 (70.6%) cases. Multiple procedures were performed in 29 (17.8%) patients 24 (14.7%) patients had two procedures and 5 (3%) patients had 3 procedures). At the end of the follow-up, 121 (74.2%) patients were stone free.

Perioperative complications occurred in 16 (9.8%) patients (5 pyelonephritis, 3 macroscopic hematuria, 6 pains with the necessity of grade II analgesia, and 1 urinoma). Patient and stone characteristics are reported in the Table-1.

**Table 1:** Characteristics of patients and Calculi.

		N=163 patients
Sex, male (n,%)		86 (54.6 %)
Age (y)		52.8±17
BMI (cm/kg^2^)		26.2±5.9
Multiple stones (n,%)		73 (44.7%)
Diameter of the largest stone		12.9±5.7
Cumulative stone diameter (mm)		15±8.6
**Stone composition**		
	Calcium oxalate monohydrate (n,%)	56 (34%)
	Calcium oxalate dehydrate (n,%)	8 (4.9%)
	Uric acid (n,%)	13 (8%)
	Carbapatite (n,%)	27 (16.6%)
	Unclear (n,%)	59 (36.1%)
Preoperative stenting (n,%)		46 (63.8%)
Mean operation time (min)		96.4±40.78
Ureteral Access Sheath (n, %)		144 (88.3%)
Postoperative ureteral JJ stent		115 (70.6%)
Multiple procedures (n, %)		29 (17.8%)
Stone free rate (n,%)		121 (74.2%)
Follow-up (months)		15.5±11.5

#### Analysis of the immediate post-operative renal function

The median GFR after procedure was 89.45 mL/min/1,73m^2^. An acute kidney injury, defined as any GFR less than 15 mL/minute/1.73m^2^, occurred in one (0.06%) patient and no patients needed dialysis. Significant renal function change occurred in 18 cases (11%) with 13 (7.9%) amelioration and 5 (3%) deterioration.

#### Analysis of long-term renal function

After a mean follow-up of 15.5±11.5 months, 14 (8.6%), 8 (4.9%) and 0 (0%) patients presented 3a, 3b and 4 CKD stages, respectively. The median GFR after procedure was 84.9±24.4 mL/ min/1.73m^2^. There was no significant difference with the preoperative median GFR (p=0.675). A change towards a worse or a better CKD group was observed in 8 (4.9%) and 23 (14.1%) cases, respectively (Table-2).

**Table 2:** Analysis of renal function after ureteroscopy and intracorporeal lithotripsy for intrarenal stones.

		Preoperative	Postoperative	p
**CKD group, n (%)**				
	GFR>60 mL/min/1.73m^2^	128 (78.5%)	141 (86.5%)	0.003
	45≤GFR<59 mL/min/1.73m^2^	27 (16.6%)	14 (8.6%)	0.018
	30≤GFR<45 mL/min/1.73m^2^	7 (4.3%)	8 (4.9%)	0.529
	15≤GFR<30 mL/min/1.73m^2^	1 (0.6%)	0 (0%)	0.319
	GFR<15 mL/min/1.73m^2^	0 (0%)	0 (0%)	1
Change to a worse CKD group			8 (4.9%)	
Change to a better CKD group			23 (14.1%)	
Mean GFR (mL/min/1.73m^2^)		84.30±26.2	84.90±24.4	0.675

#### Analysis of predicting factors of renal function changes

The univariate Cox regression analysis showed only two significant factors for renal deterioration: presence of multiple procedures and pre-existing chronic kidney disease (p=0.023; HR=5.4 and p=0.011; HR=6.8 respectively). In multivariate analysis these factors did not remain as predictive (p=0.156; HR=3.12 and p=0.054; HR=4.7) (Table-3). None of the analysed factors were predictive of renal function amelioration (Table-4).

**Table 3:** Univariable and multivariable Cox regression analysis of predicting factors for renal function deterioration in 163 patients treated by retrograde flexible ureteroscopy and intracorporeal lithotripsy for intrarenal stones.

		Univariable		Multivariable	
	p value	HR (CI)	p value		HR (IC)
Female gender (versus male)	0.109	0.26 (0.05-1.3)	-	-	
Age (continuous)	0.069	1.043 (0.99-1.09)	-	-	
Multiple stones (vs unique stone)	0.3	3.1 (0.36-25.6)	-	-	
Cumulative stone diameter (continuous)	0.587	0.96 (0.819-1.120)	-	-	
Stone free (vs stone persistence)	0.088	0.26 (0.06-1.22)	-	-	
Multiple procedures	0.023	5.4 (1.2 - 23.1)	0.156	3.12 (0.65-15)	
Preoperative stenting (vs. no stent)	0.117	5.4 (0.65-45.5)	-	-	
Preoperative CKD group >1	0.011	6.8 (1.6-30.4)	0.054	4.7 (0.97-23.4)	
Operation time (continuous)	0.522	1.005 (0.98-1.02)	-	-	
Postoperative double pigtail stent (vs ureteral stent)	0.773	1.2 (0.2-7.3)	-	-	
Postoperative pyelonephritis	0.416	2.5 (0.27-23.3)			

HR=Hazard ratio; CI=Confidence interval

**Table 4:** Univariable Cox regression analysis of predicting factors for renal function amelioration in 163 patients treated by retrograde flexible ureteroscopy and intracorporeal lithotripsy for intrarenal stones.

	Univariable	
	p value	HR (CI)
Female gender (versus male)	0.251	1.7 (0.7- 4.1)
Age (continuous)	0.051	1.028 (0.99- 1.056)
Multiple stones (vs unique stone)	0.546	0.75 (0.29-1.9)
Cumulative stone diameter (continuous)	0.445	0.97 (0.9-1.05)
Stone free (vs stone persistence)	0.121	2.1 (0.82-5.35)
Multiple procedures	0.546	0.75 (0.295 - 1.9)
Preoperative stenting (vs. no stent)	0.765	0.872 (0.35-2.1)
Operation time (continuous)	0.311	1.006 (0.99-1.02)
Postoperative double pigtail stent (vs ureteral stent)	0.087	3. (0.85-10.6)

HR=Hazard ratio; CI=Confidence interval

## DISCUSSION

In this study, we intended to evaluate the incidence of CKD after RIRS for stone management. A key finding of this study is that RIRS seemed to have a small impact on kidney function and was associated with 14.1% of long-term improvement.

Ureteroscopy is recommended by the European Association of Urology guidelines as a first–line treatment for proximal ureteral stones greater than 1 cm, but they do not recommend this procedure as a first-line therapy for intra-renal stones (7). This is essentially due to the efficiency and the non-invasive nature of SWL. Moreover, complications of RIRS are often arguments for its detractors. Nonetheless, if infection or ureteral injuries have been widely reported and studied, the renal functional outcomes after RIRS are unknown (8–10).

As we know, the use of pressurized water during RIRS leads to a dilatation of renal cavities. However, the consequences of these high intrarenal pressures have not been studied. What we do know is that excessive renal pressure is the main factor of kidney destruction during acute obstructions. Tubular function is threatened by acute excessive urinary pressure. The renal tubular cells stretched by the hydrostatic pressure leads to a tubular interstitial inflammation with macrophages proliferation and myoblasts accumulation. Alteration of the tubular cells associated with macrophages and myoblasts infiltration lead to the production of cytokines and growth factors which are responsible for renal tubular cell apoptosis. This results in chronic obstructive nephropathy with tubular atrophy and loss of nephrons which are replaced by interstitial fibrosis (11–13).

Concerning evaluation of the GFR, we focused on pre-operative, early post-operative and long-term blood creatinine level measurement. Even if the mean GFR before and after RIRS was not significantly different, we found a trend towards GFR improvement. This can be explained by numerous factors. The true prevalence of obstructive nephropathy is unknown but it is known that nephrolithiasis duration, subsequent urinary tract infections, and size of stones are factors that influence renal function (14–16). As a consequence, removing these factors may reasonably influence GFR positively.

In our study, multiple procedures appear as predicting factors of renal function loss in univariate analysis but did not stay significant in multivariate analysis. This could be explained by the effect of RIRS on the kidney but also by the fact that patients with multiple procedures often have larger stones and more advanced nephrolithiasis disease. Interestingly, operative time was not a predictive factor of renal function loss. In this context, kidney function did not seem to be a good argument for limiting the RIRS procedure time. Moreover, if a UAS is known to decrease intra-renal pressure, we did not find any influence on post-operative renal functional outcomes (17). The univariate cox regression analysis also showed that pre-existing renal dysfunction was another factor correlated with renal function. This result is not surprising considering that renal insufficiency is known as a risk factor for intra and post-operative complications in general surgery and, more particularly, in renal surgery (18). Only a 63 years old man with previous stage 3b CKD (37 mL/min) had an acute renal failure (13 mL/min) after the second procedure (120 min) for multiple renal stones (cumulative stone diameter of 15mm). He did not need hemodyalisis. Three months after RIRS and with hyperhydratation, his renal function was 28 mL/min.

Considering the superior number of patients changing to a better CKD group, we researched predictive factors of renal function improvement but no significant result clearly appears. However, our result confirmed that removal of a stone improves postoperative renal function (3).

To our knowledge, there is no study assessing the renal functional outcomes after flexible ureteroscopy. Nevertheless, this point is essential in the therapeutic decision for the management of kidney stone diseases. PNL is considered as a more invasive procedure and is indicated for larger stone diameters (more than 2 cm). For these larger stones, if RIRS is performed, multiple procedures might be required, whereas usually only a single session is required for PNL. Moreover, PNL is known to have a good functional outcome even on solitary kidneys (3, 19). A kidney functional approach in stone management is primordial. Indeed, nephrolithiasis is considered as a chronic disease, and patients often undergo multiple procedures. Thus, the management of nephrolithiasis must take into consideration the kidney functional outcomes especially in fragile patients. According to our data, we believe that the improvements of laser and flexible ureteroscope technologies raise the possibility of mini-invasive approaches.

There are several limitations to this study. First, this is a retrospective review from a single institution. The results are based on a relatively small sample size and we could not confirm that multiple procedures and preoperative kidney disease were prognostic factors of kidney function deterioration in multivariate analysis. Moreover, there might have been a confusing factor due to the contralateral compensation of the non-treated kidney for all patients with two kidneys. However, a solitary kidney model could have been more informative but it is rare and represents a very different clinical situation. Moreover, kidney function deterioration could also be linked to urolithiasis disease and it is difficult to know the part played by either natural history of the disease or RIRS. Larger cohorts and longer periods of follow-up will be necessary to consolidate these data and to confirm the identified predictive factors.

## CONCLUSIONS

After RIRS, 8 (4.9%) patients had a decrease and 23 (14.1%) patients had an improvement of their renal function. Stone management with RIRS seems to have favorable outcomes on kidney function; however, special attention should be given to patients with multiple procedures and preoperative chronic kidney disease.
